# Knowledge and Awareness of Congenital Umbilical Hernia Among the General Population of Al-Qunfudah Governorate, Saudi Arabia: A Cross-Sectional Study

**DOI:** 10.7759/cureus.114828

**Published:** 2026-08-20

**Authors:** Medhat Taha, Fuad M Alkudaysi, Sarah Y Alsayed, Danah Alfahemi, Nouf Dhaef Alassi, Majd O Albeah, Fatimah M Alsalhi, Salihah Alessa, Reham Amer Alshehri, Fatima A Alfaqih

**Affiliations:** 1 Anatomy, Umm Al-Qura University, Al-Qunfudhah, SAU; 2 Pediatrics, South Al-Qunfudhah General Hospital, Al-Qunfudhah, SAU; 3 Medicine and Surgery, Umm Al-Qura University, Al-Qunfudhah, SAU

**Keywords:** al-qunfudah, awareness, congenital hernia, cross-sectional study, knowledge, saudi arabia, umbilical hernia

## Abstract

Background: Congenital umbilical hernia is a common abdominal wall defect in newborns. It is typically asymptomatic and self-limiting, with most cases resolving spontaneously before the age of two years. Surgical intervention is indicated only when the hernia becomes symptomatic or persists beyond two years of age.

Objectives: To assess the level of knowledge and awareness regarding congenital umbilical hernia among the general population in Al-Qunfudah governorate, Saudi Arabia.

Methods: A cross-sectional study was conducted among 403 adults aged 18 years and older residing in Al-Qunfudah governorate. Data were collected using a validated, self-administered online questionnaire following informed consent. Knowledge was assessed using a composite scoring system (range: 0-14) and categorized as poor (0-6), moderate (7-10), or good (11-14). Statistical analysis was performed using Statistical Product and Service Solutions (SPSS, version 26.0; IBM SPSS Statistics for Windows, Armonk, NY), employing independent t-tests, one-way ANOVA, and chi-square tests as appropriate.

Results: The mean knowledge score was 3.9 ± 2.5 out of a maximum of 14. The majority of participants (84.1%) had poor knowledge, while 15.9% demonstrated moderate knowledge. None of the participants achieved a good knowledge score. Educational level was the only sociodemographic factor significantly associated with knowledge scores (p = 0.024), with higher educational attainment correlating with better knowledge.

Conclusion: Knowledge regarding congenital umbilical hernia among the population of Al-Qunfudah is grossly inadequate. Higher educational level is associated with better knowledge. These findings underscore the urgent need for targeted community-based educational programs to improve public awareness, facilitate early recognition, and promote timely medical consultation.

## Introduction

Congenital umbilical hernia is a common condition encountered in newborns. It is characterized by a protrusion of abdominal contents through a defect in the umbilical ring, presenting as a noticeable bulge around the navel. This bulge typically becomes more prominent when the infant cries or strains. The condition arises when the umbilical ring fails to close completely during fetal development [1].

The vast majority of affected infants remain asymptomatic. However, in cases where incarceration or strangulation occurs, symptoms such as abdominal pain, nausea, and vomiting may develop, necessitating urgent medical attention [2,3]. Diagnosis is primarily clinical, based on history and physical examination, and imaging is rarely required [1].

Epidemiologically, congenital umbilical hernia is highly prevalent, accounting for 15%-23% of newborns in the United States, affecting approximately 800,000 children annually [4]. The incidence is notably higher in certain populations, reaching up to 85% in infants of African descent and up to 75% in infants with a birth weight of less than 1.5 kg [5,6]. The condition affects males and females almost equally [7].

Management is largely conservative; most asymptomatic hernias resolve spontaneously as the umbilical ring closes. Surgical repair is reserved for symptomatic cases, complications such as incarceration or strangulation, hernias persisting beyond four years of age, those associated with genetic syndromes, or cases involving ascites or peritoneal dialysis. Importantly, the risk of recurrence is higher if surgery is performed before four years of age [3].

Despite the high prevalence of congenital umbilical hernia, data regarding public knowledge and awareness are scarce. A national study conducted in Saudi Arabia involving 680 participants reported that only 2.8% had a high level of knowledge, 10.4% had intermediate knowledge, and 86.8% had low knowledge [8]. Given the paucity of region-specific data, particularly in Al-Qunfudah governorate, this study was designed to assess the knowledge and awareness of congenital umbilical hernia among the population of this region.

Given the limited data on public awareness of congenital umbilical hernia in Al-Qunfudah governorate, this study aims to evaluate the level of knowledge and awareness regarding this condition among the general population in this region.

## Materials and methods

This was a community-based cross-sectional study utilizing an online-based self-administered questionnaire conducted in Al-Qunfudah governorate, Saudi Arabia, between January and June 2024. The study utilized both online and paper-based questionnaires distributed through social media platforms and primary healthcare centers. The study targeted adults aged 18 years and older who were residing in Al-Qunfudah governorate at the time of the study. Individuals under 18 years of age and non-residents of Al-Qunfudah governorate were excluded from participation. Additionally, healthcare professionals, including physicians, nurses, and medical students, were excluded to ensure that the findings accurately reflected the knowledge level of the general population rather than that of trained experts and to avoid overestimating the population-level knowledge. A total of 403 participants met the inclusion criteria and were enrolled in the study. The sample size was calculated using the Raosoft sample size calculator (Raosoft, Inc., Seattle, WA), assuming a 50% prevalence of adequate knowledge, a 5% margin of error, and a 95% confidence interval. The minimum required sample size was 300 participants. A total of 403 participants were ultimately enrolled.

A structured, self-administered questionnaire was developed by the authors based on a thorough literature review and expert consultation (Appendix). The questionnaire comprised three sections: demographic information, a self-assessment of knowledge, and an objective knowledge assessment. The demographic section captured age, gender, marital status, occupational status, educational level, and self-rated knowledge. The self-assessment section included a single-item question asking participants to rate their own knowledge as weak, fair, good, or excellent. The objective knowledge assessment consisted of eleven multiple-choice questions evaluating factual knowledge about congenital umbilical hernia, covering etiology, risk factors, complications, natural history, and treatment. The questionnaire was pretested on 20 participants to ensure clarity and reliability, with minor modifications made accordingly.

The scoring system was designed to yield a composite score ranging from 0 to 14, calculated as the sum of the self-assessment score (0-3) and the objective knowledge score (0-11). The scoring system consisted of a self-assessment score ranging from 0 to 3 (weak = 0, fair = 1, good = 2, excellent = 3) and an objective knowledge score ranging from 0 to 11. The objective knowledge score assigned 1 point for each correct answer and 0 for incorrect or "I don't know" responses, except for one question that carried a maximum of 2 points, bringing the total objective knowledge score to 11. These scores were combined to create a composite knowledge score ranging from 0 to 14, which was then categorized as poor (0-6 points), moderate (7-10 points), and good (11-14 points).

The reliability and internal consistency of the objective knowledge questions were assessed using Cronbach's alpha, which yielded a value of 0.72, indicating acceptable reliability. Data were entered into Microsoft Excel 2016 (Microsoft® Corp., Redmond, WA) and analyzed using Statistical Product and Service Solutions (SPSS, version 26.0; IBM SPSS Statistics for Windows, Armonk, NY). Descriptive statistics were presented as frequencies and percentages for categorical variables and means with standard deviations for continuous variables. Internal consistency was assessed using Cronbach's alpha. For inferential analysis, independent t-tests and one-way ANOVA were used to compare mean knowledge scores across demographic subgroups. Chi-square tests were used to assess associations between knowledge levels and categorical variables. A p-value of less than 0.05 was considered statistically significant.

## Results

Participant demographics

A total of 403 participants were included in the study. The demographic characteristics of the study population are summarized in Table 1. The majority of participants were aged 41 years or older (n = 157, 39.0%), female (n = 223, 55.3%), married (n = 277, 68.7%), and employed (n = 205, 50.9%). Regarding educational attainment, 55.1% (n = 222) held a bachelor's degree. When asked to self-evaluate their knowledge, 45.9% (n = 185) rated it as weak, 34.7% (n = 140) as fair, 13.4% (n = 54) as good, and only 6.0% (n = 24) as excellent.

**Table 1:** Sociodemographic characteristics of the participants (N = 403)

Parameter		Frequency (%)
Age	19-21	26 (6.5%)
	22-30	120 (29.8%)
	31-40	100 (24.8%)
	≥41	157 (39%)
Sex	Female	223 (55.3%)
	Male	180 (44.7%)
Marital status	Single	119 (29.5%)
	Married	277 (68.7%)
	Divorced	7 (1.7%)
Occupational status	Student	66 (16.4%)
	Not employed	132 (32.8%)
	Employed	205 (50.9%)
Educational level	Primary	9 (2.2%)
	Intermediate	9 (2.2%)
	Secondary	69 (17.1%)
	Diploma	78 (19.4%)
	Bachelor	222 (55.1%)
	Master	11 (2.7%)
	PhD	5 (1.2%)
Self-rated knowledge	Weak	185 (45.9%)
	Fair	140 (34.7%)
	Good	54 (13.4%)
	Excellent	24 (6.0%)

Knowledge assessment

The detailed distribution of responses to each knowledge question is presented in Table 2. The data reveal substantial knowledge gaps among the participants. While 50.6% (n = 204) correctly identified congenital umbilical hernia as a moderate condition requiring medical attention, a striking 57.1% (n = 230) did not know the contents of the hernia sac. Similarly, high proportions of participants responded "I don't know" to questions regarding risk factors and outcomes. For instance, 69.0% (n = 278) were unaware of the association with Down syndrome, 70.7% (n = 285) did not know the link with hypothyroidism, and 52.9% (n = 213) were uncertain about the possibility of recurrence after treatment.

**Table 2:** Distribution of responses to knowledge questions (N = 403)

Question Response		Frequency (%)
How do you perceive congenital umbilical hernia?	Serious, requires emergency intervention	80 (19.9%)
	Mild, does not require doctor visit	29 (7.2%)
	Mild, but require doctor visit	90 (22.3%)
	Moderate, require doctor visit	204 (50.6%)
Most ‎common content inside the ‎bulge?‎	Liquids	21 (5.2%)
	Part of the intestine	135 (33.5%)
	Air	17 (4.2%)
	I don’t know	230 (57.1%)
Have you noticed umbilical swelling in children aged 1 month-12 years?‎	Yes	63 (15.6%)
	No	227 (56.3%)
	I don't know	113 (28%)
Is congenital umbilical ‎hernia common in children?‎	Yes	135 (33.5%)
	No	99 (24.6%)
	I don't know	169 (41.9%)
More common in children ‎with Down syndrome?‎	Yes	67 (16.6%)
	No	58 (14.4%)
	I don't know	278 (69%)
Higher risk with ‎hypothyroidism?‎	Yes	62 (15.4%)
	No	56 (13.9%)
	I don't know	285 (70.7%)
Higher risk with chronic ‎cough?‎	Yes	223 (55.3%)
	No	35 (8.7%)
	I don't know	145 (36%)
Are complications common?‎	Yes	120 (29.8%)
	No	80 (19.9%)
	I don't know	203 (50.4%)
Can it ‎resolve spontaneously?‎	Yes	104 (25.8%)
	No	120 (29.8%)
	I don't know	179 (44.4%)
Is surgery always ‎required?‎	Yes	168 (41.7%)
	No	95 (23.6%)
	I don't know	140 (34.7%)
Can it recur ‎after treatment?‎	Yes	142 (35.2%)
	No	48 (11.9%)
	I don't know	213 (52.9%)

There was a marked discrepancy between participants' self-rated knowledge and their actual objective scores. As shown in Table 1, while 45.9% of participants rated their own knowledge as "weak," a far greater proportion (84.1%) were objectively classified as having "poor" knowledge based on the composite scoring system. This indicates that laypersons may not accurately perceive their own lack of medical knowledge.

Knowledge scores

The overall knowledge scores of the participants are summarized in Table 3. The mean composite knowledge score was 3.9 ± 2.5 out of a maximum of 14, with scores ranging from 0 to 10. The mean score was extremely low, with (n = 339, 84.1%) falling into the poor knowledge category, while only 15.9% (n = 64) achieved a moderate level. Notably, no participant attained a good knowledge score.

**Table 3:** Distribution of knowledge scores

Statistic	Value
Score Range (out of 14)	0-10
Mean ± SD	3.9 ± 2.5
Knowledge Category	
Poor (0-6)	339 (84.1%)
Moderate (7-10)	64 (15.9%)
Good (11-14)	0 (0%)

Bivariate analysis

The bivariate analysis comparing mean knowledge scores across different demographic variables is displayed in Table 4. Among all variables assessed, only educational level demonstrated a statistically significant association with knowledge scores (p = 0.024). Participants with a PhD had the highest mean score (5 ± 4), while those with primary education had the lowest (3 ± 2). However, the small sample size in the PhD group (n = 5) limited the power for detailed subgroup analysis. No significant associations were observed for age, sex, marital status, occupational status, or self-rated knowledge.

**Table 4:** Comparison of mean knowledge scores by demographic variables Statistically significant (p < 0.05).

Variable	Category	Knowledge score (Mean ± SD)	Test statistic	P-value
Age	19-21	3 ± 2	F = 2.5	0.057
	22-30	4 ± 2		
	31-40	4 ± 3		
	≥41	4 ± 3		
Sex	Female	4 ± 2	t = 1.3	0.207
	Male	4 ± 3		
Marital status	Single	4 ± 2	F = 0.2	0.790
	Married	4 ± 3		
	Divorced	4 ± 3		
Occupational status	Student	3 ± 2	F = 2.1	0.127
	Not employed	4 ± 2		
	Employed	4 ± 3		
Educational level	Primary	3 ± 2	F = 2.5	0.024*
	Intermediate	4 ± 3		
	Secondary	4 ± 2		
	Diploma	3 ± 3		
	Bachelor	4 ± 2		
	Master	4 ± 3		
	PhD	5 ± 4		
Self-rated knowledge	Weak	4 ± 3	F = 1.0	0.380
	Fair	4 ± 3		
	Good	4 ± 2		
	Excellent	3 ± 2		

## Discussion

This study aimed to assess the level of knowledge and awareness regarding congenital umbilical hernia among the general population of Al-Qunfudah governorate, Saudi Arabia. Our findings reveal a critically low level of awareness, with 84.1% of participants exhibiting poor knowledge and a mean score of only 3.9 out of 14. No participant achieved a good knowledge score.

These results are consistent with previous studies conducted in Saudi Arabia. Abokrecha et al. [8] reported that 86.8% of their nationwide sample had low knowledge regarding congenital umbilical hernia. Similarly, Assakran et al. [2] found that 64.3% of parents in the Qassim region had poor knowledge about congenital hernias, with only 2.3% demonstrating good knowledge. Helal et al. [9] reported comparable findings in the Madinah region, where 46.8% of parents had poor knowledge and 44.1% had average knowledge. The consistency of these findings across different regions of Saudi Arabia suggests a widespread public health education gap regarding this common congenital condition.

In contrast, studies that assessed knowledge of hernias in general (rather than specifically congenital umbilical hernia) reported higher awareness levels [10-12]. This discrepancy underscores the importance of distinguishing between general hernia awareness and condition-specific knowledge. Public health campaigns often address common hernias in adults, while less attention is directed toward congenital conditions, which may explain the particularly low awareness observed in our study. Furthermore, the often asymptomatic and self-limiting nature of congenital umbilical hernia may lead individuals to underestimate its clinical significance, reducing their motivation to seek accurate information.

Educational level was the only factor significantly associated with knowledge in our study (p = 0.024). This finding aligns with Assakran et al. [2], who also reported a significant association between educational attainment and hernia knowledge. Higher education likely provides individuals with better health literacy skills, including the ability to access, understand, and utilize health information effectively. However, our findings differ from those of Abokrecha et al. [8], who found significant associations with age and healthcare profession, and from Alsaigh et al. [11], who reported better knowledge among females, younger individuals, and those with higher education. These discrepancies may be attributed to differences in study populations, sample sizes, geographic scope, and the specific knowledge assessment tools used.

The profoundly poor knowledge observed in our study may be explained by several interconnected factors. First, public health campaigns in Saudi Arabia have historically concentrated on prevalent adult conditions such as diabetes, hypertension, and cardiovascular diseases, with congenital anomalies like umbilical hernia receiving considerably less attention from health authorities. Second, the typically asymptomatic and self-limiting nature of early umbilical hernias may lead parents and caregivers to underestimate their clinical significance, thereby reducing their motivation to seek accurate information or consult healthcare providers. Third, a significant portion of the population may depend on unverified online sources and social media platforms for health information, which often disseminate misleading content and create a false sense of reassurance regarding conditions perceived as minor. Finally, the lack of integration of basic pediatric surgical conditions into school health curricula may contribute to the persistence of knowledge deficits across generations. Educational interventions should specifically target individuals with lower educational attainment. We recommend implementing school-based health education programs and utilizing social media to disseminate simple, localized infographics about warning signs, particularly in primary healthcare centers in rural areas of Al-Qunfudah.

This study has several limitations that should be acknowledged. First, the study was conducted exclusively in Al-Qunfudah governorate, which may limit the applicability of findings to other regions of Saudi Arabia with different demographic and socioeconomic characteristics. Second, the use of convenience sampling, primarily through online platforms, may have introduced selection bias, potentially excluding individuals with limited internet access or those who are less technologically literate. Third, reliance on self-administered questionnaires may be subject to recall and response biases, including social desirability bias where participants may overestimate their knowledge. Fourth, the cross-sectional design captures knowledge at a single time point and does not assess changes over time or the effectiveness of educational interventions. Despite these limitations, the study provides valuable baseline data on public awareness in this region and highlights the urgent need for targeted educational initiatives.

## Conclusions

This study demonstrates a significant deficit in knowledge and awareness regarding congenital umbilical hernia among the population of Al-Qunfudah governorate. Higher educational attainment was significantly associated with better knowledge, while other demographic factors did not show a significant relationship.

These findings highlight the critical need for targeted, community-based educational programs and public health campaigns to improve awareness of congenital umbilical hernia. Such initiatives should employ simple, clear language and leverage multiple platforms, including social media, primary healthcare centers, and schools, to reach all segments of the population effectively. Particular attention should be directed toward individuals with lower educational attainment, who may benefit most from accessible health education materials. Early recognition and timely medical consultation are essential to prevent complications and optimize outcomes for affected children. We recommend that future research explore the effectiveness of such educational interventions, evaluate the impact of school-based health education programs, and expand the scope of investigation to other regions of Saudi Arabia to obtain a more comprehensive understanding of national awareness levels.

## Appendices

English version of the questionnaire

Questionnaire on ‌Knowledge Towards Congenital Umbilical‌ ‌Hernia‌ ‌Among‌ ‌Adults‌ ‌in‌ ‌AL‌ ‌Qunfudah,‌ ‌Saudi‌ ‌Arabia

We are a research team from the College of Medicine at Umm Al-Qura University - College of Medicine, Al-Qunfudhah District Branch. We invite you to participate in this questionnaire, which aims to assess the level of knowledge about congenital umbilical hernia among Adults in Al-Qunfudhah District and the surrounding centers. Please kindly answer the questions asked and provide the researcher with your valuable opinions by choosing the answer that you think is appropriate. If you choose to participate in this survey, you will have to complete this questionnaire, which contains questions in the form of multiple options. Filling out this questionnaire will take approximately two minutes to complete.

Please note that all the questions asked in this questionnaire are for the purposes of scientific research and to develop the level of health in the governorate, and that your answers will be surrounded by complete confidentiality and utmost scientific care.

Your participation in this survey is optional, and you have the right to withdraw from this study at any time and without explanation of the reasons for your decision.

Thank you for your cooperation and your response.

**Table 5:** Sociodemographic characteristics

Sociodemographic characteristics						
1-Age	19-21	22-30	31-40	41 or more		
2-Sex	Male	Female				
3-Marital status	Single	Married	Divorced			
4-Occupational status	Student	Not employed	Employed			
5-Educational level	Primary	Secondary	Diploma	Bachelor	Master	PhD

**Table 6:** Awareness and knowledge

Awareness and knowledge				
1-How will you evaluate your knowledge about congenital umbilical hernia?	Weak	Fair	Good	Excellent
2-What do you know about congenital umbilical hernia?	It is a mild condition and does not require a visit to the doctor.	It is a mild condition but needs to see a doctor.	It is a moderate condition and needs to see a doctor.	It is a serious condition that requires emergency medical intervention
3-In your opinion, what is the most common content found inside the bulge of the umbilical hernia?	Part of the intestine	Air	Liquid	I don’t know
4-Have you ever noticed any children exhibiting swelling in the umbilical area when they were between 1 month and 12 years old?	Yes	No	I don’t know	
5-Do you think congenital umbilical hernia is common in children?	Yes	No	I don’t know	
6-Do you think congenital umbilical hernia is more common in children with Down syndrome?	Yes	No	I don’t know	
7-Do you think children with hypothyroidism has high chance to develop congenital umbilical hernia?	Yes	No	I don’t know	
8-Do you think children with chronic cough has high chance to develop congenital umbilical hernia?	Yes	No	I don’t know	
9-Do you think congenital umbilical hernia complications are common?	Yes	No	I don’t know	
10-Can congenital umbilical hernia resolve on its own?	Yes	No	I don’t know	
11-Do you think surgical repair is always required to treat congenital umbilical hernia?	Yes	No	I don’t know	
12-Can congenital umbilical hernia recur after treatment?	Yes	No	I don’t know	

Arabic version of the questionnaire

استبيان قياس المعرفة والوعي بالفتق السري الخلقي ‌بين سكان محافظة القنفذة

نحن فريق بحثي من كلية الطب بجامعة أم القرى - كلية طب فرع محافظة القنفذة ندعوك للمشاركة في هذا الاستبيان الذي يهدف إلى دراسة وقياس المعرفة والوعي بالفتق السري الخلقي بين المواطنين في محافظة القنفذة والمراكز المحيطة بها، نرجو التكرم والإجابة على الأسئلة المطروحة وتزويد الباحث بآرائكم القيمة من خلال اختيار الإجابة التي ترونها ملائمة، إذا اخترت المشاركة في هذا الاستبيان سيتعين عليك إكمال هذا الاستبيان والذي يحتوي على أسئلة في صيغة خيارات متعددة، سيستغرق تعبئة هذا الاستبيان دقيقتين تقريبا لإكماله .

يرجى العلم أن جميع الأسئلة المطروحة ضمن هذا الاستبيان لأغراض البحث العلمي ولتطوير مستوى الصحة في المحافظة وأن إجاباتكم ستكون محاطة بالسرية الكاملة والعناية العلمية الفائقة.

مشاركتك في هذا الاستبيان اختيارية، ولديك الحق في الانسحاب من هذه الدراسة في أي وقت وبدون توضيح للأسباب قرارك.

شكرا لتعاونكم وحسن استجابتكم....

**Table 7:** البيانات الديموغرافية

البيانات الديموغرافية							
١-العمر	١٩-٢١	٢٢-٣٠	٣١-٤٠	٤١ أو أكثر			
٢-الجنس	ذكر	أنثى					
٣-الحالة الاجتماعية	أعزب	متزوج	مطلق				
٤-الحالة الوظيفية	طالب	لا يعمل	يعمل				
٥-المستوى التعليمي	ابتدائي	متوسط	ثانوي	دبلوم	بكالوريوس	ماجستير	دكتوراه

**Table 8:** الوعي والمعرفة

الوعي والمعرفة				
١-كيف تقيم مستوى معلوماتك حول الفتق السري الخلقي؟	ضعيف	متوسط	جيد	ممتاز
٢-ماذا تعرف عن الفتق السري الخلقي؟	هي حالة خفيفة ولا تتطلب زيارة الطبيب.	هي حالة خفيفة، ولكن تحتاج زيارة الطبيب.	هي حالة متوسطة وتتطلب زيارة الطبيب.	هي حالة خطيرة تتطلب تدخل طبي طارئ.
٣-بحسب معرفتك، ما الذي يوجد بداخل الفتق السري الخلقي؟	جزء من الامعاء	هواء	سوائل	لا أعلم
٤-هل سبق لك أن لاحظت ظهور تورم في منطقة السرة لدى أطفالك عند عمر (١ شهر) و (١٢سنة)؟	نعم	لا	لا أعلم	
٥-هل تعتقد أن الفتق السري الخلقي شائع عند الأطفال؟	نعم	لا	لا أعلم	
٦-هل تعتقد أن الفتق السري الخلقي أكثر شيوعاً عند الأطفال المصابين بمتلازمة داون؟	نعم	لا	لا أعلم	
٧-هل تعتقد أن الأطفال الذين يعانون من قصور(خمول)الغدة الدرقية لديهم فرصة عالية للإصابة بالفتق السري الخلقي؟	نعم	لا	لا أعلم	
٨-هل تعتقد أن الأطفال الذين يعانون من السعال(الكحه)المزمنة لديهم فرصة عالية للإصابة بالفتق السري الخلقي؟	نعم	لا	لا أعلم	
٩-هل تعتقد أن مضاعفات الفتق السري شائعة؟	نعم	لا	لا أعلم	
١٠-بحسب معرفتك هل يمكن أن يختفي الفتق السري من تلقاء نفسه؟	نعم	لا	لا أعلم	
١١-هل تعتقد ان التدخل الجراحي ضروري دائماً لعلاج الفتق السري الخلقي؟	نعم	لا	لا أعلم	
١٢-بحسب معرفتك هل يمكن أن يتكرر فتق السرة الخلقي بعد العلاج؟	نعم	لا	لا أعلم	
